# *N*-Acyl Amino Acids: Metabolism, Molecular Targets, and Role in Biological Processes

**DOI:** 10.3390/biom9120822

**Published:** 2019-12-03

**Authors:** Natalia Battista, Monica Bari, Tiziana Bisogno

**Affiliations:** 1 Faculty of Bioscience and Technology for Food, Agriculture and Environment, University of Teramo, 64100 Teramo, Italy; 2 Department of Experimental Medicine, University of Rome Tor Vergata, 00133 Rome, Italy; 3 Endocannabinoid Research Group, Institute of Translational Pharmacology, National Research Council, 00133 Rome, Italy

**Keywords:** *N*-acyl amino acids, *N*-acyl glycines, *N*-acyl serines

## Abstract

The lipid signal is becoming increasingly crowded as increasingly fatty acid amide derivatives are being identified and considered relevant therapeutic targets. The identification of *N*-arachidonoyl-ethanolamine as endogenous ligand of cannabinoid type-1 and type-2 receptors as well as the development of different–omics technologies have the merit to have led to the discovery of a huge number of naturally occurring *N*-acyl-amines. Among those mediators, *N*-acyl amino acids, chemically related to the endocannabinoids and belonging to the complex lipid signaling system now known as endocannabinoidome, have been rapidly growing for their therapeutic potential. Here, we review the current knowledge of the mechanisms for the biosynthesis and inactivation of the *N*-acyl amino acids, as well as the various molecular targets for some of the *N*-acyl amino acids described so far.

## 1. Introduction

*N*-acyl amino acids (NAAAs) are an important family of endogenous signaling molecules in which an amide bond covalently links an amino acid to the acyl moiety of a long-chain fatty acid. Although lipid containing amino acids, some of them produced in response to certain stress conditions, were identified in different bacterial membranes, little is known about their specific molecular functions [1,2]. In addition to NAAAs, amino acid derivatives of short- and/or medium-chain fatty acid with relevant involvement in both physiological and/or pathological conditions have been reported in literature [3,4,5].

The discovery of *N*-arachidonoyl-ethanolamine (AEA) as one of the endogenous ligand able to bind with different affinity cannabinoid type-1 (CB_1_) and type-2 (CB_2_) receptors, with which NAAAs share chemical structure and same biologic activities, let to a renewed scientific interest toward these compounds. Indeed, some NAAAs were initially synthesized for structure-activity relationship studies to optimize the CB_1_ binding and later, identified as naturally occurring in bovine and rat brain [6,7]. Subsequently, the development of functional proteomic technology for enzymes allowed the identification of a new class of metabolites significantly elevated in mice in which the gene encoding for AEA-hydrolyzing enzyme fatty acid amide hydrolase (FAAH) has been ablated [8]. These compounds were identified as amides between very long-chain fatty acids and sulfur-containing amino acid, taurine, (*N*-acyl taurines, NATs) [8]. Later, a targeted lipidomics approach unveiled the existence of a large family of naturally occurring NAAAs including derivatives of proteogenic α-amino acids, taurine and γ-aminobutyric acid (GABA) [9,10,11]. Even though the number of NAAAs has been rapidly growing in recent years and they have been emerging as important family of endogenous signaling molecules, only a limited amount of research has been reported on some of these compounds and their physiological role(s) is not known. Among these, it should be mentioned that several *N*-acyl alanines (Ala) exhibited antiproliferative effect in vitro [12] as well as *N*-oleoyl phenylalanine (Phe) regulated energy homeostasis [13]. Moreover, stearoyl derivates of tyrosine (Tyr), serine (Ser), and threonine (Thr) exerted neuroprotection activity [14] and *N*-linoleoyl Tyr, by activating CB_2_ receptor, protected against transient cerebral ischemia [15].

The purpose of this article is to overview NAAAs to provide information as complete and as updated as possible regarding their biosynthetic pathways, physiological role, and biological activity. Therefore, in this review we summarize literature data on the most widely studied member of NAAA family, i.e., *N*-acyl glycines (NAGlys) and NASers, to underscore that the understanding of biochemical and molecular mechanisms, underlying their pharmacological actions, may serve to find way to properly exploit their therapeutic potential.

## 2. Metabolic Pathways for NAAAs

**Figure 1:**
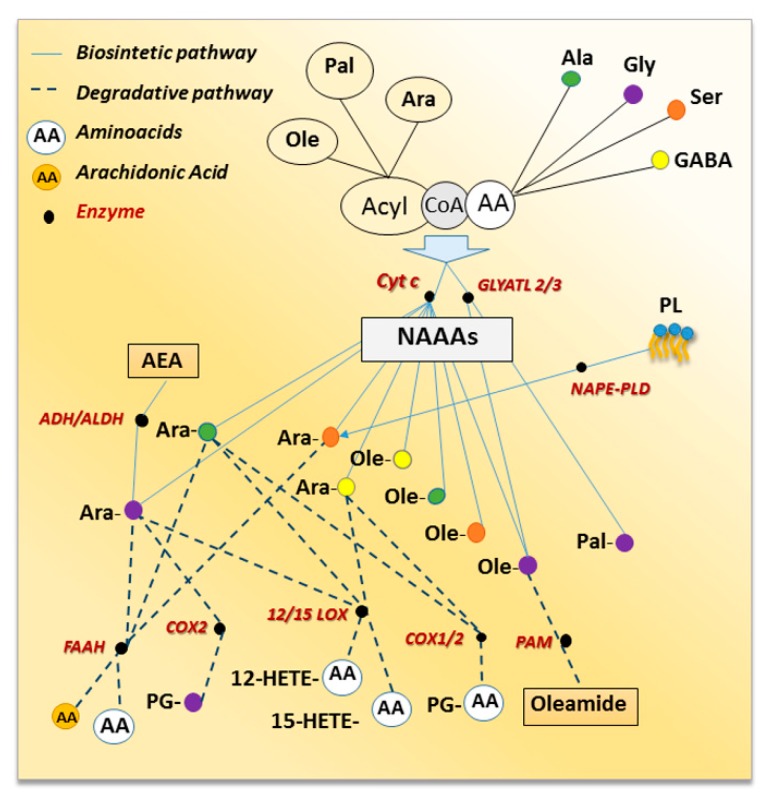
Schematic network of NAAAs metabolic routes, which shows interaction between biosynthetic (solid lines) and degradative (dot lines) pathways. Enzymes are black circles; white color represents all the amino acids while colored circles are the amino acids detailed in the test.

Any amino acid, theoretically, might be able to form an amide with any fatty acid, thereby generating hundreds of novel bioactive lipids, via metabolic pathways that are still not completely understood [16]. Little is known about the biosynthesis of NAAAs except for NAGlys, for which the anabolic processes have been investigated (Figure 1). One possible hypothesis involves the direct condensation of the acyl moiety, both free fatty acid or CoenzymeA (CoA) derivative, with glycine [7,17]. Indeed, the enzymatic action of cytochrome c catalyzing the synthesis of *N*-arachidonoyl glycine (NAraGly) from glycine and Ara-CoA in presence or not of hydrogen peroxide was reported [7,17]. This pathway accounts also for *N*-oleoyl glycine (NOleGly) formation [18] as well as for other NAraAAs as cytochrome c-dependent formation of NAraSer, NAraAla, and *N*-arachidonoyl γ-aminobutyric acid (NAraGABA) from Ara-CoA and the respective amino acids were described [7,17]. In addition, the amide bond formation between medium- and long-chain acyl-CoAs and glycine may be also mediated by glycine *N*-acyltransferase-like 2 (GLYATL2) and 3 (GLYATL3) described in human [19] and in mouse cells [20] respectively. Indeed, GLYATL2 catalyzed the synthesis of NAGly from acyl-CoA and glycine preferring oleoyl-CoA as a substrate [19] and, reduced levels of *N*-palmitoyl glycine (NPalGly) and NOleGly were detected in siRNA knockdown of GLYATL3-N18TG2 cells [20]. A second pathway suggested that NAraGly might be generated by oxidative metabolism of the corresponding *N*-arachidonoyl-ethanolamine via the sequential enzymatic reaction of alcohol dehydrogenase (ADH) and aldehyde dehydrogenase [21,22]. This was supported by the identification of human ADH7 catalyzing the oxidative metabolism of AEA to NAraGly through the formation of *N*-arachidonoyl glycinal as intermediate [23]. It seems clear that further research effort is needed to fully understand the biosynthesis of the NAAAs and more importantly, whether these routes occur in distinct tissues or cells, and again whether they are mutually exclusive or if under different physio/pathological conditions one route may prevail on the other. It should be also noted that some NAAAs, e.g., NAraSer, might be, theoretically, produced by phospholipid hydrolysis following an identical biosynthetic pathway to that responsible for AEA formation. Lipidomics profile of mouse brain in which the gene encoding for AEA biosynthetic enzyme (NAPE-specific phospholipase D, NAPE-PLD) has been ablated, documented alteration in NAAA levels [24]. Indeed, with respect of NAraAAs the authors observed that NAraAla and NAraPhe decreased in the brainstem and striatum, respectively, while NAraGly increased in the hypothalamus, cerebellum, cortex, and thalamus. The brain region-dependent effects of NAPE-PLD knockout (KO) was also documented for NAraSer levels that decreased in the cerebellum and increased in the midbrain, as well as for NAraTyr that decreased in the thalamus and increased in the cortex [24].

Regarding NAAAs inactivation, since these compounds have chemical features in common with AEA, it was expected that they might share some of its catabolic fate (Figure 1). Indeed, FAAH also catalyzes the hydrolysis of other long-chain *N*-acyl-amides including NATs and NAAAs. Several NAAAs were synthesized and their potencies as FAAH inhibitors were evaluated [25]. The relative potency of NAraGly, Ala, valine (Val), leucine (Leu), isoleucine (Ile), glutamate (Glu), glutamine (Gln), aspartate (Asp), Tyr, and Phe was assessed on FAAH preparations obtained from human, rat, and mouse and, was found to be dependent on the mammalian species. Among the NAraAAs tested, only NAraGly, Ile and Ala inhibited FAAH and NAraGly behaved as a competitive inhibitor [25]. Moreover, lipidomics analyses of NAAAs, obtained from the combination of six fatty acids (i.e., palmitic, stearic, oleic, linoleic, arachidonic and docosahexaenoic acid) with proteinogenic (i.e., Ala, Gly, Leu, Met, Phe, Pro, Ser, Trp, Tyr and Val) or non-proteinogenic (GABA) amino acids, were performed in different brain areas of FAAH KO mice [10]. Curiously, except for increased NAraSer content, all the other NAraAAs levels decreased in FAAH KO mice [24]. In addition, increased levels of NPal, *N*-stearoyl, and NOle derivatives of Ala, Gly and Ser were also detected in all brain regions analyzed [24].

Growing evidence indicates that, just like AEA, some NAraAAs because of the presence of arachidonic acid, are also substrates to most of the oxidative metabolic pathways that lead to eicosanoid biosynthesis. Indeed, NAraGly, NAraAla, and NAraGABA are hydroxylated to the corresponding 12- and 15-hydroxyeicosatetraenoic (HETE) derivatives by both 12- and 15-lipoxygenases (LOX) at rates comparable to arachidonic acid [26]. Cyclooxygenase-2 (COX-2), but not COX-1, metabolized NAraGly to prostaglandin PG-glycine (PG-Gly) and site-directed mutagenesis studies identified Arg-513 as the residue mainly responsible for COX-2 selectivity towards NAraGly [19]. Both NAraAla and NAraGABA were slightly oxidized by COX-2 and, unlike NAraGly, they were also metabolized by COX-1 though the rate of conversion was even lower than for COX-2 [26,27]. Finally, peptidylglycine α-amidating monooxygenase (PAM), an enzyme involved in the production of peptide amides, catalyzed the oxidative cleavage of NOleGly to the primary fatty acid amide, oleamide [28,29]. It should be noted that PAM catalytic action might be considered either one of the possible mechanisms for NAGlys inactivation or the last step required for primary fatty acid amide formation as PAM gene knockdown strongly reduced primary acyl-amide and increased NAGly levels in N18TG2 cells [20].

## 3. *N*-Acyl Glycines

NAGlys are widely distributed throughout the central nervous system and other mammalian tissues, where they play preeminent roles in cell physiology and exhibit interesting pharmacological properties. Although an accurate quantification of their tissue levels is still missing, the endogenous concentration of these molecules ranges from high nM to low μM values. Based on its chemical similarity with AEA, NAraGly might be considered the most representative of this family and is certainly the most studied one. Analgesic and anti-inflammatory activities as well as vasorelaxation and inhibition of T-type calcium channels are some of the features of NAraGly recently summarized in a comprehensive review [30]. Different G-protein coupled receptors (GPCRs) account for NAraGly biological functions (see Table 1) and FAAH and COX-2 might affect its endogenous levels and bioactivity. Among GPCRs, NAraGly shows high affinity for the orphan GPR18 [31], GPR55 [32] and GPR92 [33], whereas it does not bind to CB_1_ [6] or CB_2_ [31]. Although GPR18 binding assays have not been validated yet, several pieces of evidence supported the involvement of GPR18 in mediating some physiological and functional activities of NAraGly including calcium mobilization [31], control of cell apoptosis [34] and migration [35], anti-inflammatory action [36], neuropathic pain [37], diurnal regulation of intraocular pressure [38] and microglial-neuronal communication [39]. It was also recently reported that in an in vitro model of organotypic hippocampal slice cultures, NAraGly induced neuroprotection through the activation of GPR18 as its effect was partially reverted by GPR18 receptor antagonists [40]. In addition, NAraGly modified primary microglia cell morphology, from amoeboid to ramified shape, but did not affect neither cell size nor motility [40]. Moreover, it induced astrocyte activation as assessed by the increasing of glial fibrillary acidic protein (GFAP) expression levels and decreased PI3K/Akt pathway in glial cells, but not in neurons [40]. However, the role of NAraGly as GPR18 agonist is still debated, since controversial results were obtained by screening NAraGly in the β-arrestin PathHunter™ system [41] as well as in a native neuronal system [42]. Recently, Console-Bram and co-workers, by using a stable cell line expressing hemagglutinin (HA) incorporated into the *N*-terminus of the human GPR55 cDNA (HAGPR55/CHO), reported that NAraGly behaved as GPR55 agonist [32]. Indeed, NAraGly in a dose-dependent manner increased intracellular calcium levels and mitogen-activated protein kinase (MAPK) signaling, and these effects were selectively blocked by specific GPR55 antagonist [32]. Finally, molecular modeling and site-directed mutagenesis studies revealed specific amino acidic residues required to GPR92 to bind to both hydrocarbon chain and glycine moiety of NAraGly [33]. NAraGly reduced allodynia in an animal model of neuropathic pain [37] and, by activating GPR92, increased calcium mobilization, inositol phosphate production and cAMP levels in small diameter dorsal root ganglion (DRG) neurons [33]. NAraGly functionally interacts with GABA_A_ receptor [43] and behaves as a positive allosteric modulator, probably sharing a common binding site with the endocannabinoid 2-arachidonoylglycerol [43]. Moreover, it has been reported that NAraGly can also interact with glycine receptors in a dichotomous mode: potentiating α1 and inhibiting α2 and α3-containing glycine receptors [44]. In addition, NAraGly acts as non-competitive inhibitor of the glycine transporter, GLYT2a, and has a small inhibitory effect on the GABA transporter, GAT1 [45]. On this basis, it can be speculated that the interaction of NAraGly with GLYT2a might be responsible for analgesia in the hotplate test [46] by likely modulating the glycinergic neurotransmission [45]. Again, NAraGly exhibited spinal analgesic actions by enhancing inhibitory glycinergic and decreasing excitatory NMDA-mediated synaptic transmission [47]. It should be also recalled the ability of NAraGly to affect ion currents through T-type calcium [48,49] and small conductance potassium channels (SK_Ca_) [38]. Finally, NAraGly inhibited high voltage activated Cav3.2 calcium currents strongly ameliorating the thermal analgesia in vivo, and counteracted endothelin-1 (ET-1)-induced vasoconstriction in retinal arterioles by triggering an endothelium-dependent signaling mechanism that involves SK_Ca_ channels [38]. NOleGly, another important member of the NAGly family detected at high concentration in mammalian tissues and cells as well as in *Drosophila melanogaster* [11,20,50], activates the peroxisome proliferation proliferator-activated receptor α (PPAR-α), CB_1_, and inhibits GLYT2 and paraoxonase [51,52,53]. Although the functional activities of NOleGly are still largely unknown, its predominant role in the regulation of behavioral actions, such as body temperature and motor activity, in the control of physiological processes, such as energy homeostasis and food, has been recently reported [52,54,55]. NOleGly ameliorated withdrawal-associated behaviors and prevented nicotine addiction in vivo [56]. The anti-reward effect, assessed in the conditioned place preference (CPP) paradigm, required PPAR-α activation as was reverted by PPAR-α antagonist administration [56]. Moreover, the capability of NOleGly to interfere with the aversive properties associated with acute naloxone-precipitated morphine withdrawal (MWD) and the rewarding effects of morphine in Sprague–Dawley rats has been investigated [57]. NOleGly impacted MWD and the CB_1_ antagonist, AM251, but not the PPARα antagonist, MK886, prevented NOleGly effect suggesting a CB_1_ receptor-mediating action. Altogether, these data point out NOleGly as a potential player in combating the high tobacco smoking rate and in protecting against the negative effects of drugs of abuse. Finally, NOleGly was reported to be active in reducing cell proliferation in vitro assay using the RAW 267.4 mouse macrophage cells [58], although in vivo studies are needed to validate this molecule as a promising drug candidate in cancer therapy.

NPalGly was identified in murine cells and tissues [50] and human fluids [68]. Notably, it was detected in *Drosophila melanogaster* that also expressed acyl derivatives of Val, Leu, Ala, Tryr, Phe, Ser and GABA [11,69]. The bioactivity of this molecule is still poor studied and the lack of significant effects of NPalGly as antiviral against Sendai virus fusion to liposomes [70] as well as anti-inflammatory agent in the phorbol ester-induced mouse ear edema model have been reported [58]. It has been speculated that NPalGly, might be considered a putative ligand of transient receptor potential canonical 5 (TRPC5) [67,71]. Indeed, NPalGly is high expressed in rat skin and spinal cord and inhibited heat-evoked firing of nociceptive neurons in rat dorsal horn, activated calcium influx in DRG cells and stimulated NO production. These effects were reverted by TRP-channel blocker. Given the marked similarity in the NPalGly activity profile and TRPC5 response, it might be possible that NPalGly activates TRPC5 [67,71]. Of note, no further data have confirmed this theory.

Recently, a urinary metabonomic study showed a significant increase of NPalGly in primary dysmenorrhea women suggesting that this elmiric acid, together with other metabolites, could be a potential biomarker in clinical diagnosis and treatment of this gynecological disorder [68]. In addition, a global metabolite profiling analysis identified NPalGly as the major palmitate-derivative produced in HER2/neu-positive breast cancer cells, suggesting NPalGly implication in the systemic responses to the tumor as well as in the etiology of breast cancer [72].

## 4. *N*-Acyl-Serines

Little is known regarding the subfamily of NASers and they might still be considered endogenous orphan lipid mediators.

NAraSer was isolated ten years ago from bovine brain [59] and, except for its negative interaction with high voltage activated Cav3.2 calcium currents [73], to date no specific receptor-mediating NAraSer actions have been identified. Since from its first identification, NAraSer exhibited no affinity toward CB_1_, CB_2_ or TRPV1 [59], although some of the effects of NAraSer were similar to those reported for the classical endocannabinoids [74]. In particular, as reported in Table 1, NAraSer caused endothelium-dependent arterial vasodilatation and stimulated phosphorylation of p44/42 MAPK and protein kinase PKB/Akt in cultured endothelial cells, effects that were not dependent on CB_1_/CB_2_ activation [59]. Similar profile of actions of NAraSer and abnormal cannabidiol (Abn-CBD) allowed to hypothesize a common mechanism of action for these compounds suggesting that both bind to GPR18 [59]. Later, Kino and co-workers showed that NAraSer, in vitro, participated in cytoskeleton reorganization and intracellular signaling by acting as modulator of MAPK, Akt, JNK, and c-JUN phosphorylation via CB_1_, CB_2_ and TRPV1 activation [62]. NAraSer might act as a pro-angiogenic lipid and might affect vascular repairs by regulating endothelial growth and migration, via modulation of vascular endothelial growth factor C [75]. Of note, NAraSer-induced signal transduction and endothelial functions were partly reduced in GPR55 knockdown mice [75]. Several members of NASer family, including NAra, NPal, and NOle derivatives, have been tested as neuroprotective agents after traumatic brain injury (TBI) [61,63,64]. While NAraSer, as with 2-AG, caused improvement of neurological severity score as well as reduction of lesion volume, NPalSer only slightly affected neurological behavior and did not modify the typical neuropathology following TBI. Any improvement in neurological severity score nor on the cognitive function were reported for NOleSer [64]. It should be also noted that NAraSer increased proliferation and decreased differentiation of neural progenitor cells (NPC) into astrocytes and neurons after TBI. The neurogenic properties of NAraSer, in NPC, isolated from embryonic cortical tissue and grown as neurospheres, were impacted by CB_1_, CB_2_ and TRPV1 receptors [61,63,64]. Finally, NAraSer exhibited anti-microbial effect related to biofilm-associated antibiotic resistance. It impaired drug-resistant pathogens by inhibiting biofilm formation and reducing metabolic activity of mature biofilm, this might be considered a promising alternative to antibiotic therapeutics against biofilm-associated infections [60].

In the last few years, published data suggested NOleSer as new potential therapeutic against bone diseases. Indeed NOleSer, which naturally occurs in bone, increased osteoblast proliferation and reduced osteoclast survival by modulating Erk1/2 phosphorylation in vitro as well as affected bone volume density in vivo [65]. However, in a mouse ovariectomized model for postmenopausal osteoporosis, NOleSer, as novel antiosteoporotic drug, reduced bone resorption and enhanced bone formation [65]. Finally, NOleSer serum levels decreased in individuals with the rare genetic disorder Prader Willi syndrome (PWS), characterized by low bone mineral density and increased risk of bone fractures secondary to osteoporosis, and were positively correlated with reduced bone density. Moreover, reduced NOleSer levels were detected in mice KO for the maternally imprinted gene in the PWS critical region, Magel2, causing osteoblast dysfunction, increased osteoclastogenesis and osteoclast activity [66].

## 5. Conclusions

Different technologies, such as proteomics, lipidomics, bioinformatics as well as systems biology approaches let to the identification of many NAAAs in the mammalian body. In this article, we have attempted to provide an extensive and comprehensive summary of 1) biosynthesis, mechanism of action and inactivation of NAAAs and 2) the role of NAGlys and NASers in biological processes. For the experimental data described here, it is evident that considerable progress has been made in this research field, although we are still far from fully appreciating the physiological importance of NAAAs and, more importantly, their involvement in pathological conditions. It is crucial to be established, for example, how and following which stimuli they might be produced or whether their endogenous levels might be modulated by age, environmental factors, epigenetic changes as well as nutrition. Indeed, since both fatty acids and amino acids are essential diet elements, it is realistic to hypothesize that NAAA tissue levels might be modulated by food. Again, NAAAs, which share with endocannabinoids both inactivating enzymes and molecular targets, have been considered “endocannabinoidome” mediators [76], but further investigations are required to verify whether this huge variety of compounds interact leading to joint beneficial actions or obstruct each other causing unwanted side effects.

## Figures and Tables

**Table 1:** Main effects of NAGlys and NASers mediated by different GPCRs and other receptors.

	Glycine	Serine
*N*-arachidonoyl	Calcium mobilization [31] Cancer cell proliferation [58] Cell apoptosis and migration [34,35] Cell morphology [40] Inflammation [36] Intraocular pressure [38] Microglial-neuronal communication [39] Neuropathic pain [45] Thermal analgesia [46]	Angiogenesis [59] Anti-microbial effect [60] Cell proliferation [61] Cytoskeleton reorganization [62] Neuroprotection [61,63,64]
*N*-Oleoyl	Body and motor activities [54] Cancer cell proliferation [58] Energy homeostasis [52] Food intake [55] Nicotine addiction [56]	Bone metabolism [65,66]
*N*-Palmitoyl	Calcium mobilization [67] Primary dysmenorrhea [68]	Neuroprotection [64]
